# Familial Mediterranean Fever: an unusual cause of liver disease

**DOI:** 10.1186/s13052-019-0712-0

**Published:** 2019-09-18

**Authors:** Maria Cristina Maggio, Maria Castiglia, Giovanni Corsello

**Affiliations:** 1 Department of Health Promotion Sciences Maternal and Infantile Care, Internal Medicine and Medical Specialities “G. D’Alessandro”, University of Palermo, Palermo, Italy; 2 Paediatric Radiodiagnostic Unit, Children Hospital “G. Di Cristina”, ARNAS, Palermo, Italy

**Keywords:** Familial Mediterranean fever, Liver disease, Colchicine, Canakinumab

## Abstract

**Background:**

Familial Mediterranean Fever is an autoinflammatory disease typically expressed with recurrent attacks of fever, serositis, aphthous stomatitis, rash. Only a few reports describe the association with hepatic involvement.

**Case presentation:**

We describe the clinical case of a child affected, since the age of 1 year, by recurrent fever, aphthous stomatitis, rash, arthralgia, associated with abdominal pain, vomiting, lymphadenopathy. The diagnosis of Familial Mediterranean Fever was confirmed by the genetic study of MEFV gene; the homozygous mutation M694 V in exon was documented. A partial control of attacks was obtained with colchicine. The child continued to manifest only recurrent episodes of abdominal pain without fever, however serum amyloid A persisted high, in association with enhanced levels of CRP, AST and ALT (1.5 x n.v.). The dosage of colchicine was increased step by step and the patient achieved a better control of symptoms and biochemical parameters. However, the patient frequently needed an increase in the dose of colchicine, suggesting the possible usefulness of anti-interleukin-1 beta treatment.

**Conclusions:**

The unusual presentation of Familial Mediterranean Fever with liver disease suggests the role of inflammasome in hepatic inflammation. Colchicine controls systemic inflammation in most of the patients; however, subclinical inflammation can persist in some of them and can manifest with increased levels of CRP, ESR, serum amyloid A also in attack-free intervals.

## Background

Familial Mediterranean Fever (FMF) is an autoinflammatory disease typically expressed with recurrent attacks of fever, serositis leading to abdominal, thoracic or articular pain, aphthous stomatitis, erysipelas-like erythema [1]. Only a few reports in the international literature describe the association with hepatic involvement, documented by ultrasound and increased levels of AST, ALT, gamma-GT, C-reactive protein (CRP), erythrocyte sedimentation rate (ESR). In these cases, an accurate differential diagnosis is needed to exclude other immune-mediated or infectious diseases mimicking a liver disease [2, 3].

## Case presentation

We report on a 10.6-year-old child affected, since the age of 1 year, by recurrent fever, aphthous stomatitis, rash, arthralgia, associated with abdominal pain, vomiting, lymphadenopathy. Recurrence was every 15–30 days. Serum amyloid A (SAA) levels were 33 mg/l, CRP was 24.8 mg/dl, ESR was 86, AST and ALT were 1.5 x n.v.. Though, he showed a height gain corresponding to age (stature: 132.2 cm: - 1.55 SDS; weight: 27 kg: - 1.72 SDS), he did not showed obesity with a Body Mass Index of 15.5 (− 1.3 SDS).

The diagnosis of FMF was considered for the clinical presentation of the child [1] and confirmed at the age of 6 years by the genetic study of MEFV gene. The patient showed a homozygous mutation of exon 10: M694 V. Therefore, he started the treatment with colchicine at the dosage of 0.03 mg/kg/day. The grandfather died for renal insufficiency. The parents did not refer recurrent fever and/or other symptoms associated with FMF, and they did not agree to be investigated genetically for FMF.

A partial control of attacks was gained by colchicine (however, recurrent episodes of abdominal pain, without fever persisted) and increased levels of SAA persisted. For these reasons, the dosage of colchicine was gradually increased to 0.05 mg/kg/day, following the regular weight gain during growth, with a good control of biochemical parameters. SAA showed high levels (33–223.8 mg/l: n.v.: < 6.4) in some phases of the follow up, in correlation with the dosage of colchicine. However, the normalization was achieved after the progressive increase of the dose.

**Fig. 1:**
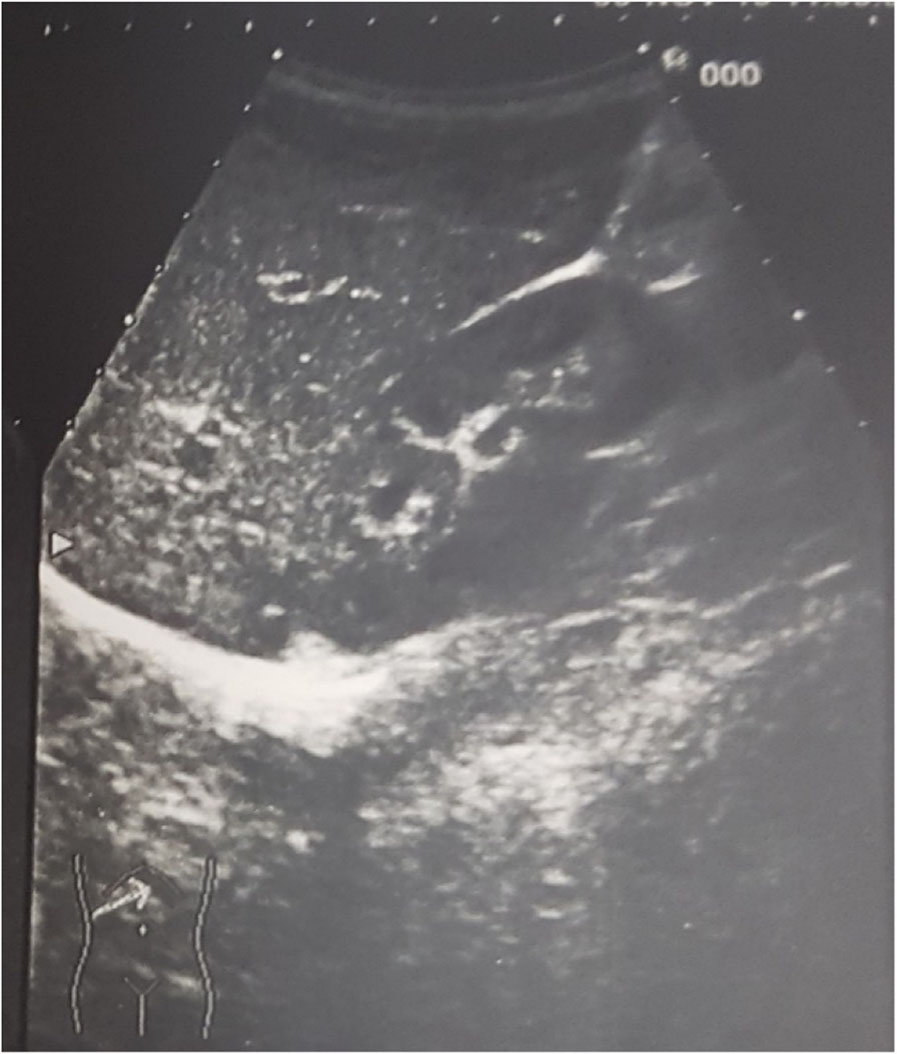
Abdomen ultrasound of the patient, showing a "starry-sky liver"

Abdominal ultrasound documented lymphadenopathy and an echography pattern of “starry sky liver” (Fig. 1) consensual with the phases of the disease characterized by a significant increase of SAA and a mild increase of AST, ALT (1.5 x n.v.). A better control of the disease with increased doses of colchicine, however, allowed to normalize SAA, AST, ALT levels and liver ultrasound pattern. These data confirm that liver involvement was not secondary to colchicine toxicity. Furthermore, the patient maintained a low BMI, excluding obesity as a cause of hepatopathy. He did not assume other drugs further than colchicine that could contribute to the increase of transaminases or induce hepatopathy.

Currently, SAA is 1.77 mg/l; CRP: 0.18; AST: 24; ALT: 28; urinalysis is in the normal range without microalbuminuria (Table 1).

**Table 1:** Outcome of biochemical parameters of the patient

	Colchicine mg/day (mg/kg/day)	SAA (mg/l)	CRP (mg/dl)	ESR	AST	ALT	GGT	microalbuminuria (n.v.: 0–20 mg/l)
at the admittance		33	24.8	86	82	68	21	9198
At 6.9 years	0.75 (0.03)	32.4	0.23	17	26	32	20	9400
At 8.2 years	1.25 (0.05)	223.8	7	42	76	62	33	4
At 10.6 years	1.5 (0.05)	1.77	0.18	11	24	28	12	10.5

## Discussion and conclusions

We described this case for the unusual presentation of FMF with fever, rash, aphthous stomatitis, liver disease, not associated to other conditions, as infections, drugs intolerance [2], autoimmune disease, celiac disease [4]. The described case underscores the chance that MEFV mutations provide hepatic inflammation, as seen in this child, by the inflammasome involvement.

The reported case of a male child with FMF, presenting recurrent episodes of hepatitis (spontaneously resolved) during fever attacks or disease phases with a low control by colchicine, highlight the role of an appropriate colchicine dosage in the control of inflammation. In this view, the inflammatory role of the disease in the liver involvement is confirmed by the consensual increase of SAA, AST, ALT. Therapeutic trial with increased doses of colchicine was successful, confuting the hypothesis of the possible role of colchicine toxicity in this case.

Cryptogenic liver disease was described in patients with FMF only in a few studies [5, 6]. However, the cases reported in the international literature showed more frequently the M694 V mutation, especially in homozygous state.

Some studies reported the safety profile of IL-1 blockers from real life [7]. We discussed with the parents and the patient the opportunity to shift treatment to canakinumab, a fully human anti- interleukin (IL)-1 beta monoclonal antibody, to better control the inflammatory state of the patient and the symptoms.

Canakinumab has not been started yet, because at the last visit SAA levels were in the normal range and the patient was asymptomatic. However, the opportunity to shift the treatment to canakinumab for this patient could be the optimal choice to minimize the inflammatory state and to control the risk of amyloidosis, improving long-term prognosis and quality of life.

## Data Availability

Materials and data of the patient are included in the medical records of the patient.

## Abbreviations

CRP: C-reactive protein
ESR: Erythrocyte sedimentation rate
FMF: Familial Mediterranean Fever
IL-1: Interleukin-1
SAA: Serum amyloid A
